# Cognition and Brain Activation in Response to Various Doses of Caffeine: A Near-Infrared Spectroscopy Study

**DOI:** 10.3389/fpsyg.2020.01393

**Published:** 2020-07-03

**Authors:** Bin Zhang, Ying Liu, Xiaochun Wang, Yuqin Deng, Xinyan Zheng

**Affiliations:** ^1^Shanghai University of Sport, Shanghai, China; ^2^School of Sports Science, Nantong University, Nantong, China

**Keywords:** caffeine, different doses, cognition, brain activation, near-infrared spectroscopy

## Abstract

Caffeine, which is widely used for enhancing athletic performance, has been suggested to have a positive impact on cognition via stimulating the brain. However, no study published to date has explored the effects of different doses of caffeine ingestion on brain activation via cortical hemodynamics. The purpose of the present crossover, double-blind study was to investigate the effects of low, moderate, and high doses of caffeine ingestion on cognitive performance and brain activation. Ten healthy male subjects ingested placebo or caffeine (3, 6, or 9 mg/kg body mass). The effects of each treatment condition were evaluated by Stroop tasks before and 60 min after the ingestion of caffeine. Reaction time (RT) and accuracy of responses to congruent and incongruent stimuli were assessed. As an index of brain activation with cognition, levels of oxygenated hemoglobin (HbO) were measured via near-infrared spectroscopy. A 4 × 2 mixed ANOVA revealed that there were significant interaction effects for RT in both incongruent and congruent conditions (*P* < 0.01, *P*η^2^ = 0.384; *P* < 0.05, *P*η^2^ = 0.259; and *P* < 0.05, *P*η^2^ = 0.309). Both 3 and 6 mg/kg of caffeine ingestion significantly decreased RT to incongruent stimuli. The only dose of caffeine to decrease RT in response to congruent stimuli was 3 mg/kg. None of the doses of caffeine administered affected accuracy of responses to incongruent or congruent stimuli. Under the congruent stimulus condition, ingestion of 3 mg/kg of caffeine significantly increased mean HbO in the dorsolateral prefrontal cortex, frontal pole area, ventrolateral prefrontal cortex (*P* < 0.01, *P*η^2^ = 0.319; *P* < 0.05, *P*η^2^ = 0.263; and *P* < 0.05, *P*η^2^ = 0.259, respectively). None of the doses of caffeine investigated affected HbO under the incongruent stimulus condition. Ingestion of low-dose caffeine has greater effects on cognition and brain activation than moderate and high doses of caffeine, suggesting that low-dose caffeine may be a selective supplement in enhancing executive function and prefrontal activities.

## Introduction

Caffeine is widely used by athletes for improving exercise performance. Administration of 3–13 mg/kg body mass caffeine increases exercise performance during intensive running or cycling by 20–50% (Sökmen et al., 2008). Graham and Spriet (1995) investigated the effects of low, moderate, and high doses of caffeine on prolonged exercise capacity. They found that ingestion of 3 or 6 mg/kg of caffeine improved time to exhaustion, whereas 9 mg/kg of caffeine did not. There results indicated that the effect of low-dose caffeine ingestion had the similar ergogenic effect as moderate dose, which could improve physical ability. Excellence in sport performance requires not only physical and motor capabilities but also sensory–cognitive skills (Moscatelli et al., 2016). However, to our knowledge, no study examined the effects of low, moderate, and high doses of caffeine on cognition until now.

Stimulation of the central nervous system (CNS) has been proposed to explain caffeine’s ergogenic effects (Kalmar and Cafarelli, 2004). Caffeine acts as a central stimulant and enhances cognitive and psychomotor functioning, particularly during mental and physical fatigue, through effects that enhance alertness and vigilance. These findings suggest that the exercise performance-enhancing effects of caffeine stem from the compound’s ability to alter CNS function (Hogervorst et al., 2008).

The action of caffeine on the brain suggests an effect on cognitive performance. Cognition includes executive functioning (EF), decision making, and creativity. Executive functioning is important during exercise and can be affected by prolonged exercise (Yanagisawa et al., 2010). Reports in the scientific literature present inconsistent findings in relation to the effects of caffeine ingestion on the Stroop task performance, a measure of executive function. Some studies involving cognitive inhibition or interference conditions report faster or potential fast reaction times (RTs) with the use of caffeine (Hasenfratz and Bättig, 1992; Kenemans et al., 1999; Hogervorst et al., 2008; Dixit et al., 2012; Dodd et al., 2015; Souissi et al., 2019), whereas others report no change at all (Edwards et al., 1996; Bottoms et al., 2013). Differences in outcomes between studies may be related to the sensitivity of the cognitive tests used or the dose of caffeine administered, and more studies need to examine effects of caffeine on cognition.

The effects of caffeine on cognition may be related to the enhancement of brain activation. Early studies postulated that the effects of caffeine on brain activation depend on the complex interaction of neuronal and vascular responses. These responses may vary among brain regions, introducing an additional layer of complexity (Laurienti et al., 2003; Koppestaetter et al., 2010). Caffeine acts as a non-adenosine receptor antagonist. It blocks adenosine receptors and excites neuro-stimulants (Dunwiddie and Masino, 2001). Moreover, caffeine acts as a vasoconstrictor via blocking adenosine 2A and 2B receptors, resulting in decreased cerebral blood flow (CBF) (Laurienti et al., 2002; Pelligrion et al., 2010). The interaction of caffeine with neural and vascular systems has direct effects on neural connectivity during resting states as well as cognitive activation (Haller et al., 2014).

Brain activation is measured using neuroimaging techniques such as functional magnetic resonance imaging (fMRI) and near-infrared spectroscopy (NIRS). Using blood oxygen level-dependent (BOLD) fMRI, Diukova et al. (2012) found that caffeine ingestion increased activity in the frontal cortex. The BOLD signal is a complex functional measurement of changes in neural activity, oxygen metabolism, cerebral blood volume, and CBF (Buxton et al., 2004). The balance between blood levels of oxygenated hemoglobin (HbO) and deoxygenated hemoglobin (HbR) is a critical determinant of the BOLD response. Levels of HbO and HbR may be measured with NIRS to investigate components underlying the BOLD response. NIRS studies that evaluated the responses of participants on various tests of cognition have reported conflicting results for the effects of caffeine on HbO and HbR (Niioka and Sasaki, 2003; Higashi et al., 2004; Heilbronner et al., 2015).

To our knowledge, no NIRS-based study published to date has explored the effects of low, moderate, and high doses of caffeine on brain activation during cognitive tasks. The purpose of the present study was to investigate the effects of various doses of caffeine ingestion on brain activation and cognitive performance. We hypothesized that low-dose caffeine ingestion had similar effects as moderate dose, which could improve executive functioning and brain activation.

## Materials and Methods

### Subjects

Ten healthy, non-smoking male subjects (age 20 ± 1 year, height 1.73 ± 0.20 m, and weight 70.5 ± 4.8 kg) participated in this study. They were non-users (ingesting < 50 mg of caffeine/day). The sample size used was calculated by G-Power software [effect size (ES) = 0.15, power = 0.80; Faul et al., 2007]. Subjects were required to visit the laboratory with an empty belly and to abstain from drinking beverages containing caffeine and from use of other psychoactive substances or medication for at least 24 h before every experimental trial. All subjects were fully informed of the nature and possible risks of the study. After that, written informed consent was obtained from all subjects before study enrollment. The study followed the ethical guidelines of the Declaration of Helsinki and was approved by the local ethics committee at Shanghai University in Sport, Shanghai, China (No. 2016008).

### Protocol

Subjects visited the laboratory four times, at the same time of day. When participants arrived in the dimly lit room where experiments were to be conducted, they were seated in a comfortable chair in front of a computer monitor. After the NIRS optode grid had been positioned on the subjects’ head, they performed the Stroop familiarization trial. In order to obtain baseline measurements of performance on the Stroop task, each subject sat quietly for 5 min and watched a black screen. At that point, the NIRS recording was paused, and participants ingested capsules containing placebo (calcium carbonate; CON) or 3 mg/kg (CAF3), 6 mg/kg (CAF6), or 9 mg/kg (CAF9) of caffeine with 200 ml of water (PRE). After a 60-min delay, during which the optode grid remained in place, participants once again performed the same Stroop task (POST). The crossover, double-blind design was used in the present study. All subjects completed all experiment conditions, which were separated by 1 week to ensure drug washout period.

### Drugs

Caffeine hydrate (Wako Pure Chemical Industries, Ltd., Osaka, Japan) and calcium carbonate, which are white powder, were used in this study. The dosage of each condition was calculated according to the weight. In the CON condition, 9 mg/kg of calcium carbonate was put into three red capsules. In the CAF3 condition, both 3 mg/kg of caffeine and 6 mg/kg of calcium carbonate were put into three red capsules. In the CAF6 condition, both 6 mg/kg of caffeine and 3 mg/kg of calcium carbonate were put into three red capsules. In the CAF9 condition, 9 mg/kg of caffeine was put into three red capsules. In this way, researchers and subjects could not identify caffeine according to the appearance and taste of the capsule.

### Stroop Task

The Stroop task is widely used to evaluate selective attention, cognitive flexibility, and processing speed (Pauw et al., 2015). It was programmed and performed on E-prime 1.0 software (Psychology Software Tools, Pittsburgh, PA, United States). Each trial was displayed as follows: a fixed cross in the center of the screen for 500 ms and a stimulus duration for 500 ms. There were two kinds of stimuli in current study: congruent and incongruent conditions. The congruent condition is composed of three Chinese color words (i.e., 绿 for green, 蓝 for blue, and 红 for red), whose color was the same to the meaning of the color words (e.g., “green” word was presented in green). And the incongruent condition consisted of the same three-color words, whose color was completely different from the meaning of the color words (e.g., “green” words were presented in blue or red). Subjects were required to figure out the presenting color of each word by using the numeric keypad as the response apparatus. And the numeric keypad featured digits “1,” “2,” and “3” from left to right, which correspond to the responses of “blue,” “green,” and “red,” respectively. The subjects used the index, middle, and ring fingers of their right hand for the response of digits “1,” “2,” and “3.” According to the previous Stroop task, the RTs on figuring out the color in the incongruent condition were longer than those in the congruent condition, as the processing of word meaning in the incongruent condition interferes with the color recognition (e.g., when “green” words were presented in red, the meaning of the green word impedes the recognition of red; Stroop, 1935; Pauw et al., 2015).

Participants performed two blocks of 120 trials. Each block included 60 congruent and 60 incongruent trials, which were randomly presented. To prevent participants from anticipating a stimulus, the interval between appearance of the fixed cross and presentation of the stimulus was randomly differed between 300 and 800 ms, with the fixed inter-stimulus interval (ISI) duration of 1,500 ms. Both RT and accuracy (ACC) were recorded for further analysis.

### Hemodynamic Data Acquisition

**FIGURE 1:**
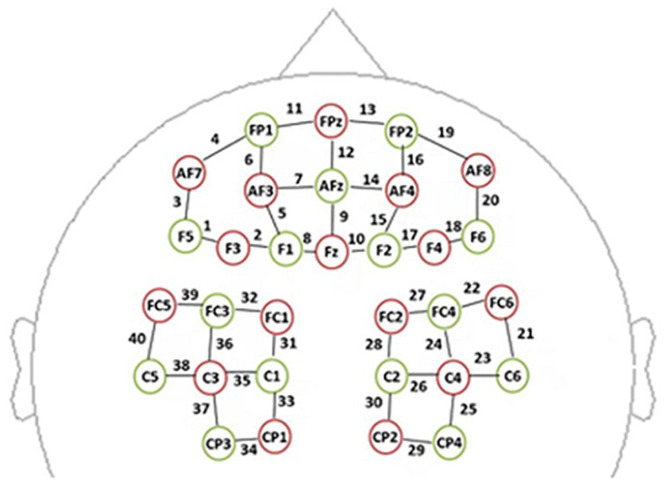
The spatial profile of functional near-infrared spectral imaging (fNIRS) probes. The red circles indicate the 16 optical sources, the green circles indicate the 15 detectors, and the black numbers (1–40) indicate fNIRS channels. The optical sources and detectors were positioned on the international 10–20 standard positions.

We used a multichannel, continuous wave, NIRS instrument (NIRScout, NIRx Medical Technologies LLC, Minneapolis, MN, United States) for monitoring hemodynamic activity during performance of the task and during the resting state. The sampling rate was 3.91 Hz. The NIRS probe included 16 dual-wavelength sources (780 and 830 nm) and 15 optical detectors, which covered the frontal and parietal areas bilaterally (Figure 1). One emitter and one detector (3 cm apart) formed a channel. Forty channels were assessed: 20 distributed throughout the frontal area and 20 distributed throughout parietal areas. The correspondence between NIRS channel locations and specific brain regions was established by Okamoto et al. (2004, 2009) and Tsuzuki et al. (2007). Probes were set according to a 10/20 electroencephalogram system, with some adjustments to ensure that each emitter was 3 cm away from its corresponding detector (Chen et al., 2018).

### Hemodynamic Data Analysis

Optical data were transformed into hemoglobin signals with arbitrary units in accordance with the modified Beer–Lambert law (Cope et al., 1988). It has been reported that HbO signals have a better signal-to-noise ratio than HbR signals (Niu et al., 2013; Schaeffer et al., 2014), so we used only HbO measurements to analysis. The HbO data were analyzed with nirsLAB software. After discontinuous shifts were removed from the time series dataset, HbO signals were bandpass-filtered between 0.01 and 0.2 Hz to remove baseline drift and physiological noise (e.g., heartbeats). Bandpass filtering was performed by a high-pass filter with a cutoff frequency of 0.01 Hz to remove low-frequency noise, such as head movement, and by a low-pass filter with a cutoff frequency of 0.2 Hz to attenuate high-frequency noise and cardiovascular artifacts (Huppert et al., 2009; Yunjie et al., 2012). Then, each subject’s HbO during a given session was calculated. Hemodynamic data were then baseline-corrected based on the mean value of all signals from each block (5 s before to 15 s after the block). The HbO data were then averaged across subjects (Chen et al., 2018).

The region of interest (ROI) channels were defined as those channels with maximal HbO. After HbO was averaged across subjects, mean HbO during the congruent and incongruent conditions was subtracted from mean HbO during the resting state. The mean difference between the single-cognitive task and resting state sessions was arranged according to descending magnitude, for each channel (Chen et al., 2018). We defined channels of interest as the top 30% of channels (greatest values). The multichannel NIRS space was transformed into traditional Montreal Neurological Institute space (Cutini et al., 2011). Channels of interest were related to three ROIs on the basis of their spatial distribution relative to the automated anatomical labeling template (Table 1). HbO values were then averaged through channels within a given ROI.

**TABLE 1:** Stroop task-related regions of interest (ROIs).

**ROI**	**Channels**	**Hemisphere**	**Location**
1	5,7,14,15	Bilateral	DLPFC
2	6,11,13,14	Bilateral	FPA
3	3,4,19,20	Bilateral	VLPFC

*DLPFC, dorsolateral prefrontal cortex; FPA, frontal pole area; VLPFC, ventrolateral prefrontal cortex.*

### Data Analysis

Statistical analyses were conducted with SPSS 20.0 software (SPSS Inc., Chicago, IL, United States). One-sample Kolmogorov–Smirnov test was used to test whether data were normally distributed. When data are not normally distributed, statistical analysis was performed on the logarithmic transformation of the data. RT, ACC and mean HbO data for all frequencies in a given ROI were subjected to repeated-measures three-way ANOVA with dose (CON/CAF3/CAF6/CAF9), time (PRE/POST), and condition (incongruent/congruent) as within subject factors to examine whether the general tendencies for the Stroop task could be reproduced in all conditions. Then alterations in RT, ACC, and averaged HbO data for all frequencies in a given ROI were subjected to 4 × 2 repeated-measures ANOVAs. For cases in which the assumption of sphericity was violated, the Greenhouse–Geisser correction was used to reduce the likelihood of a Type I error. If significant main or interaction effects were found, *post-hoc* analyses were carried out with a Bonferroni correction. Partial eta^2^ (*P*η^2^) was used as a measure of ES in the case of ANOVA. The criteria to interpret the magnitude of ES were as follows: small (*P*η^2^ = 0.01), medium (*P*η^2^ = 0.06), or large (*P*η2 = 0.14; Cohen, 1992). Data are presented as mean ± SD. Statistical significance was accepted at *P* < 0.05.

## Results

### Stroop Task Performance

The three-way ANOVA for RT and ACC demonstrated no significant interaction effects (*P* = 0.201, *P*η^2^ = 0.155; and *P* = 0.201, *P*η^2^ = 0.155, respectively). The ANOVA revealed that there were significant main effects of condition for RT (*P* < 0.001, *P*η^2^ = 0.882) and ACC (*P* < 0.01, *P*η^2^ = 0.567). These results confirmed that Stroop interference could be generally observed between the congruent and incongruent conditions.

#### Incongruent Condition

A 4 × 2 mixed ANOVA revealed that there was a significant interaction for RT [*F*(3, 27) = 5.61, *P* < 0.01, *P*η^2^ = 0.384, Table 2]. RT of 60 min after the administration was significantly decreased after treatment with 3 mg/kg (PRE, 685.43 ± 85.95 ms; POST, 649.70 ± 96.53 ms, *P* < 0.05) or 6 mg/kg (PRE, 658.83 ± 71.93 ms; POST, 624.10 ± 84.57 ms, *P* < 0.05). There was no significant difference in RT between CAF3 and CAF6. Treatment with 9 mg/kg of caffeine did not affect RT. There was no significant interaction for ACC (Table 2).

**TABLE 2:** The reaction time and accuracy rate of the Stroop test.

**Measurements**	**Condition**	**Dose (mg/kg)**	**PRE**	**95%CI**	**POST**	**95%CI**
	Incongruent (ms)	0	643.1882.80	583.95–702.42	669.1872.47	617.34–721.02
		3	685.4385.95	623.94–746.91	649.7096.53^#^	580.65–718.75
		6	658.8371.93	607.37–710.28	624.1084.56^#^	563.61–684.59
Reaction time		9	654.5493.05	587.98–721.11	649.65104.41	574.95–724.33
	Congruent (ms)	0	582.5482.05	523.84–641.23	608.8579.50	551.98–665.72
		3	580.6679.39	523.86–637.44	553.6078.32^#^	497.57–609.63
		6	576.2867.48	528.01–624.55	580.3766.12	533.07–627.66
		9	585.7467.94	537.14–634.34	574.0578.29	518.03–630.05
	Incongruent	0	0.920.07	0.87–0.96	0.950.07	0.91–1.00
		3	0.940.06	0.90–0.98	0.940.04	0.91–0.97
		6	0.950.04	0.92–0.97	0.930.06	0.89–0.98
Accuracy rate		9	0.920.05	0.89–0.96	0.950.06	0.91–0.99
	Congruent	0	0.960.03	0.93–0.98	0.960.05	0.92–1.00
		3	0.970.04	0.94–0.99	0.990.01	0.98–1.00
		6	0.980.04	0.95–1.00	0.980.02	0.97–1.00
		9	0.960.03	0.94–0.98	0.970.03	0.95–0.99

*PRE, before administration; POST, after administration. ^#^Significant PRE vs. POST (p < 0.05). Values are mean ± SD.*

#### Congruent Condition

We found a significant interaction for RT [*F*(3, 27) = 3.14, *P* < 0.05, *P*η^2^ = 0.259, Table 2]. RT had decreased significantly at 60 min after the administration of 3 mg/kg of caffeine (PRE, 580.66 ± 79.39 ms; POST, 553.60 ± 78.32 ms, *P* < 0.01). However, the administration of caffeine at doses of 6 or 9 mg/kg did not affect RT. We found no significant interaction for ACC (Table 2).

### Near-Infrared Spectroscopy

The three-way ANOVA for ROI-1 [dorsolateral prefrontal cortex (DLPFC)], ROI-2 [frontal pole area (FPA)], and ROI-3 [ventrolateral prefrontal cortex (VLPFC)] demonstrated no significant interaction effects (*P* = 0.09, *P*η^2^ = 0.230; *P* = 0.224, *P*η^2^ = 0.154; and *P* = 0.633, *P*η^2^ = 0.061, respectively). The ANOVA revealed that there was a significant main effect of condition for ROI-1 (*P* < 0.05, *P*η^2^ = 0.394).

#### Incongruent Condition

**FIGURE 2:**
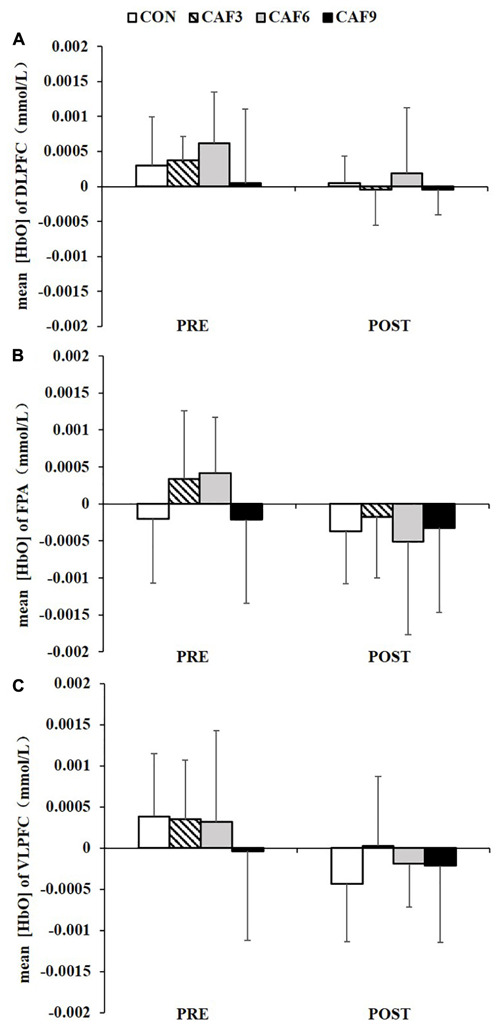
Changes in the mean [HbO] of incongruent condition in the DLPFC **(A)**, FPA **(B)**, and VLPFC **(C)**. HbO, oxygenated hemoglobin; DLPFC, dorsolateral prefrontal cortex; FPA, frontal pole area; VLPFC, ventrolateral prefrontal cortex; PRE, before administration; POST, after administration. Values are mean ± SD.

A 4 × 2 mixed ANOVA revealed that there was no significant interaction for mean HbO in ROI-1 (Figure 2A), ROI-2 (Figure 2B), or ROI-3 (Figure 2C).

#### Congruent Condition

**FIGURE 3:**
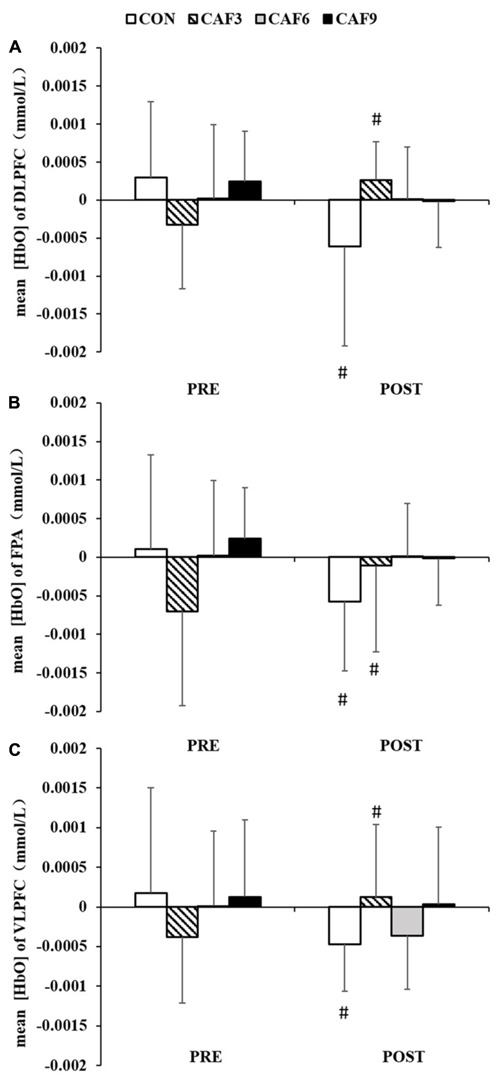
Changes in the mean [HbO] of congruent condition in DLPFC **(A)**, FPA **(B)**, and VLPFC **(C)**. HbO, oxygenated hemoglobin; DLPFC, dorsolateral prefrontal cortex; FPA, frontal pole area; VLPFC, ventrolateral prefrontal cortex; PRE, before administration; POST, after administration. ^#^Significant PRE vs. POST (*P* < 0.05). Values are mean ± SD.

We found a significant interaction for mean HbO in ROI-1 [*F*(3, 27) = 4.21, *P* < 0.01, *P*η^2^ = 0.319, Figure 3A]. In the CON group, mean HbO had significantly decreased at 60 min after administration of the placebo, as compared with baseline values. At 60 min after the administration of 3 mg/kg of caffeine, mean HbO had significantly increased. The administration of caffeine at doses of 6 or 9 mg/kg did not affect mean HbO.

A 4 × 2 mixed ANOVA revealed that there was a significant interaction for the mean HbO in ROI-2 [*F*(3, 27) = 3.21, *P* < 0.05, *P*η^2^ = 0.263, Figure 3B]. Compared with baseline values, mean HbO after 60 min showed a significant decrease in the CON group. At 60 min after the administration of 3 mg/kg of caffeine, mean HbO had significantly increased. Treatment with caffeine at doses of 6 or 9 mg/kg did not affect mean HbO.

Two-factor ANOVA of mean HbO showed significant effects of dose × time [*F*(3, 27) = 4.02, *P* < 0.05, *P*η^2^ = 0.309, Figure 3C]. Compared with baseline values, mean HbO had significantly decreased at 60 min in the CON group. At 60 min after treatment with 3 mg/kg of caffeine, mean HbO had significantly increased. Treatment with caffeine at doses of 6 or 9 mg/kg did not affect mean HbO.

## Discussion

This novel study investigated the effects of ingestion of low, moderate, or high doses of caffeine typically used by athletes on cognition and brain activation using NIRS. We found that ingestion of low doses of caffeine, but not moderate or high doses caffeine, decreased RT on the Stroop task, under the congruent and incongruent conditions, and increased mean HbO in three ROIs under the congruent condition. Ingestion of moderate doses caffeine only decreased RT on the Stroop task, under the incongruent conditions.

Low doses (≤3 mg/kg) of caffeine have been reported to improve cognition during and after strenuous exercise (Hogervorst et al., 1999, 2008). Ingestion of 3 mg/kg of caffeine, but not 6 or 9 mg/kg, decreased RT under the congruent condition on the Stroop task. This result suggests that low-dose caffeine may represent “a direct and specific ‘perceptual-motor’ speed or efficiency factor” (Nehlig, 2010). After consumption of low doses of caffeine, participants in our study showed decreased RT, accompanied by a significant decrease in interference effects. These findings are similar to those reported by Kenemans et al. (1999).

In this study, the ingestion of 6 mg/kg of caffeine decreased RT on the Stroop task under the incongruent condition. Nonetheless, the beneficial effects of 6 mg/kg of caffeine on cognitive performance are in dispute. Similar to the present study, Souissi et al. (2019) found that 6 mg/kg of caffeine ingestion improved RT and attention. Moreover, Ali et al. (2015) showed that 6 mg/kg of caffeine induced a tendency toward improvement in performance on the Stroop task among female athletes engaged in team sports. This discrepancy in results may reflect methodological differences related to the specific protocol used or the gender of the study participants. Moreover, we observed that high doses of caffeine had no effect on cognitive performance. One possible explanation for this finding is that the ingestion of high doses of caffeine induces side effects such as gastrointestinal upset, nervousness, mental confusion, and inability to focus (Graham and Spriet, 1995). Our data suggest that ingestion of low or moderate doses of caffeine ingestion decreases interference with successful performance on the Stroop task. As 3 mg/kg of caffeine improved performance on simple as well as complex cognitive tasks, we suggest that low-dose caffeine has a greater impact on cognition than moderate or high doses of caffeine.

Previous studies have reported the activation of the lateral prefrontal cortex (LPFC) upon execution of the Stroop task. Banich et al. (2000, 2001) suggested that LPFC activation may reflect interference processing and/or response inhibition. This may result in greater activation of relevant LPFC in the incongruent condition compared with the congruent condition. Milham et al. (2003) reported that during the Stroop task, the DLPFC in the LPFC is the primary region involved in the implementation of top-down attention control. Additionally, according to Krompinger and Simons (2011), the DLPFC resolves conflicts that occur during information processing of incongruent stimuli during the Stroop task. Therefore, the Stroop performance is more related to activation of the DLPFC. In the present study, we found a significant main effect of condition for the mean HbO of the DLPFC: the mean HbO in the incongruent condition was higher than in the congruent condition. These Stroop effect findings are similar to those in previous functional NIRS (fNIRS) studies, which suggested that executive functioning is associated with activation of DLPFC (Xu et al., 2017; Kujach et al., 2018). Interestingly, we found different results with previous two fNIRS studies (Xu et al., 2017; Kujach et al., 2018): the present Stroop task failed to activated the FPA and the VLPFC. But DLPFC activation in the present study is consistent with that of a previous meta-analysis review on Stroop task-related fMRI, in which FPA and VLPFC also could not be significantly activated (Nee et al., 2007; Huang et al., 2020). Thus, more fNIRS or fMRI neuroimaging studies are needed to clarify the roles of FPA and VLPFC in the Stroop task.

That caffeine improved the Stroop task performance may be related to activation of LPFC. Different from our hypothesis, caffeine ingestion failed to affect the mean HbO in the DLPFC, FPA, and VLPFC under the incongruent condition, whereas under the congruent condition, ingestion of 3 mg/kg of caffeine increased mean HbO in the DLPFC, FPA, and VLPFC. Combining the above-mentioned opposite pattern in which the mean HbO of DLPFC in the incongruent condition was higher than that in the congruent condition, indicated that mean HbO of LPFC, especially DLPFC, has been increased during Stroop-interference processing in the incongruent condition, whereas following caffeine ingestion, the significant reduction was found in the activation of LPFC. On the other hand, in the congruent condition, in which the cognitive processing is less demanding than in the incongruent condition, HbO of DLPFC has been less activated, whereas mean HbO in the DLPFC, FPA, and VLPFC was increased after the ingestion of 3 mg/kg of caffeine. These results demonstrate that under high cognitive processing, the effects of caffeine on LPFC activation have been attenuated by higher demanding processing, whereas under low cognitive tasks, the effects of caffeine on LPFC activation are more pronounced, because the congruent condition in Stroop task involved less demanding processing. The present results provide new evidences for previous studies that caffeine improvement of brain activation is induced more easily at the moment of the lowest values (Niioka and Sasaki, 2003; Souissi et al., 2019).

In the present study, under the congruent condition, no doses of caffeine ingestion affect the mean HbO. These results contrast with those of previous studies, which found that ingestion of 75 or 200 mg of caffeine was associated with decreased mean HbO on the Stroop task (Niioka and Sasaki, 2003; Dodd et al., 2015). This discrepancy in results may reflect methodological differences related to the specific protocol used. Therefore, use of the Stroop task should be standardized in future studies for investigating the effects of drugs on cerebral hemodynamic responses. Furthermore, under the congruent condition used, ingestion of 3 mg/kg of caffeine increased mean HbO in the DLPFC, FPA, and VLPFC. These results are consistent with those of a previous fMRI study, which showed that ingestion of low-dose caffeine enhanced neuro-activation in the frontal cortex (Diukova et al., 2012).

The increase in mean HbO during the Stroop task observed in this study after ingestion of low-dose caffeine may be related to an increase in regional cerebral blood volume (rCBV). Caffeine acts as an adenosine receptor antagonist and consequently as an excitatory neuro-stimulant, thus enhancing neural activity (Dunwiddie and Masino, 2001) and increasing rCBV. These findings are in line with a report by Higashi et al. (2004) that ingestion of 180 mg of caffeine maintained an increase in frontal rCBV during performance of an arithmetic task. Caffeine also regulates cerebral perfusion and acts as a vasoconstrictor, decreasing CBF via the blockade of A2A and A2B receptors (Laurienti et al., 2002; Pelligrion et al., 2010). Our observation that ingestion of low-dose caffeine increases mean HbO suggests that caffeine increases in rCBF via exciting neuro-stimulants outweigh caffeine decreases in rCBF via decreasing CBF. Along these lines, the lack of any observed effect of 6 or 9 mg/kg of caffeine on mean HbO suggests that, at these doses, the increase in rCBV may be equivalent to the decrease in CBF.

Moderate-to-high doses of caffeine administrated 1 h before and during exercise have been known to increase endurance athletic performance. In contrast, recent evidence has shown an ergogenic effect of low and extremely low doses of caffeine taken late during a period of prolonged exercise (Hogervorst et al., 1999; Jenkins et al., 2008). Furthermore, low doses of caffeine do not affect peripheral whole-body responses to exercise and are associated with few, if any, side effects; Spriet (2014) suggested that low doses of caffeine ingestion improve exercise performance In this study, we observed that ingestion of low-dose caffeine had greater effect on cognition and brain activation than had moderate and high doses, which means that low doses of caffeine have greater effect on stimulating the CNS. We suggest that ingestion of low-dose caffeine before and/or during exercise may induce more ergogenic effects than use of moderate or high doses.

### Limitation

The present study maintained a few limitations. We used G-power to estimate the sample size, and the numbers of subjects in this study met the minimum sample size requirements. However, more samples are needed in the future research so that the research results can be further verified and repeated. In the double-blind designed study, it is best to ask subjects which dose they think they ingested in each trail after completion of all groups and to outline why they identified which trial as which. However, in the present study, we did not note the responses of the subjects, so we could not assess the efficacy of blinding. Although four conditions in the present study are difficultly for participants to identify, we should value the assessment of blinding in future studies. Moreover, only Stroop task was used to measure executive function. There are other cognitive tasks on executive function, such as n-back and switching task. Therefore, more tasks are need to measure to ensure effects of various doses of caffeine ingestion on executive function in the future.

## Conclusion

The present study found that ingestion of 3 mg/kg of caffeine improved performance on the Stroop task under both the incongruent and congruent conditions and increased mean HbO under the congruent condition. Ingestion of 6 mg/kg of caffeine improved performance on the Stroop task under the incongruent condition. These results demonstrate that ingestion of low-dose caffeine has greater effects on cognition and brain activation than moderate and high doses of caffeine, suggesting that low-dose caffeine may be a selective supplement in enhancing executive function and prefrontal activities.

## Data Availability Statement

The raw data supporting the conclusions of this article will be made available by the authors, without undue reservation, to any qualified researcher.

## Ethics Statement

The study followed the ethical guidelines of the Declaration of Helsinki and was approved by the local Ethics Committee at the Shanghai University in Sport, Shanghai, China (No. 2016008). The patients/participants provided their written informed consent to participate in this study.

## Author Contributions

XZ and YD conceived and supervised the study and designed the experiments. BZ and YL carried out the experiments. YL and XW analyzed the data. BZ wrote the manuscript. All authors contributed to the article and approved the submitted version.

## Conflict of Interest

The authors declare that the research was conducted in the absence of any commercial or financial relationships that could be construed as a potential conflict of interest.
